# Using Passive Infrared Detectors to Record Group Activity and Activity in Certain Focus Areas in Fattening Pigs

**DOI:** 10.3390/ani10050792

**Published:** 2020-05-03

**Authors:** Naemi Von Jasmund, Anna Wellnitz, Manuel Stephan Krommweh, Wolfgang Büscher

**Affiliations:** Institute of Agricultural Engineering, University of Bonn, 53115 Bonn, Germany; anna.wellnitz@t-online.de (A.W.); krommweh@uni-bonn.de (M.S.K.); buescher@uni-bonn.de (W.B.)

**Keywords:** activity profile, PID, animal-based indicator, animal welfare indicator, on-farm consulting, early warning system, assessment protocol, visual assessment, ammonia

## Abstract

**Simple Summary:**

Pigs are important livestock for meat production. Because of rising demand for animal products from housing systems that enable high standards of animal welfare, solutions must be found to record and assess welfare parameters. Pigs have a wide behavioral repertoire and fixed daily routines, which offers an opportunity to detect deviations from normal behavior as a measure of welfare. In this study, we evaluated the use of passive infrared detectors (PID) for measuring group activity and activity in certain focus areas in a pen of fattening pigs. PIDs can be used to measure activity by detecting thermal changes between moving bodies and the infrared radiation they emit compared to the background. For evaluation, the data of the detectors were compared to human observation to see if the technique is able to represent the behavioral patterns of the animals correctly. The results indicate that PIDs are suitable for recording the activity of a group of pigs in a pen as well as in clearly definable areas, such as the trough. Such activity profiles obtained initial conclusions to be drawn about resting, stress, and activity phases, which can be used combined with other factors like the air temperature to assess animal welfare.

**Abstract:**

Animal behavior is an important aspect in the assessment of animal welfare. Passive infrared detectors (PID), detecting thermal changes to measure activity, have already been used to record data on the behavior of groups of animals. Within this study, the suitability of these detectors for the collection of activity profiles for focused areas is further investigated. The aim was to record the activity of a group of eleven fattening pigs in a pen, as well as the activity in the five functional areas for resting, feeding, drinking, exploration, and elimination. In order to evaluate the data obtained, the behavior was video recorded for visual assessment. In addition, relevant indoor environment parameters were recorded (ammonia, air temperature, and relative humidity). For the measurement of activity by PID, strong correlations from up to r = 0.87 (*p* < 0.01) could be found compared to visual assessment. The results indicate that activity changes during the day and activity in defined functional areas can be recorded using PIDs. These data combined with data of climate-related sensors could serve the farmer as a monitoring tool for early detection of behavioral changes or serve as partial aspect within a Weak Point Analysis within external on-farm consulting.

## 1. Introduction

Consumers are increasingly interested in the well-being of farm animals [1,2], and food quality is also defined by animal welfare and not exclusively by the quality of the end product itself [2,3]. Overall, demands of society for more species-appropriate housing conditions and for more appropriate animal welfare are increasing. In the future, this will require a continuous assessment of each housing environment and the animals living in it according to animal-welfare-relevant indicators and a precise documentation of results. Already established criteria for assessing animal welfare are often resource- and management-based. They can be used to evaluate animal welfare indirectly and by determining whether the quantity and quality of available resources are sufficient [1]. Sensors for monitoring the barn environment are, for example, air temperature and air humidity sensors or sensors for measuring gaseous pollutants such as ammonia (NH_3_). However, indicators directly related to animals such as Body Condition Score (BCS) or behavioral changes, i.e., so-called animal-based indicators, are gaining importance. They are now considered by researchers to be fundamental for evaluating welfare in its multidimensionality [2] as comprehensively as possible [1,2]. These direct indicators are already an essential part of assessment protocols such as the Welfare Quality^®^ protocol for pigs [4]. Because they are very time-consuming, they are typically used as a snapshot and as part of regular checks of the herd every few weeks or months, although continuous observation and evaluation of animal health status and behavior is considered more effective for the assessment of animal welfare.

Innovative animal-based sensor systems which try to enable the continuous and objective monitoring and evaluation of the behavior of groups of animals like pigs could use, for example, visually acquired information using appropriate sensors in combination with learning algorithms or similar applications. Thus, pixel changes between images can be used to identify animal activity in general [5] or aggressive behavior as well [6,7]. Image processing techniques based on measuring pixel intensity value between images [8] or Delaunay triangulation [9,10] can also provide information about lying behavior and potential deficiencies in temperature management. Moreover, the use of optical flow combined with modified angular histograms may indicate anomalies regarding locomotion of pigs at the slaughterhouse [11]. Image processing techniques in combination with the definition of the body contour profile of pigs allow for automatically drawing conclusions on drink nipple visits and water use [12]. One study also showed good accuracy for the automated monitoring of various behaviors such as lying, standing, feeding, drinking, and the transitioning between different behaviors, which could be classified simultaneously using depth image processing and analysis algorithms for lactating sows in farrowing crates [13].

Another approach to record behavior or rather animal activity is the use of passive infrared detectors (PID). By detecting thermal changes between moving animals and the infrared radiation emitted by them against the background, activity or movement can be measured regardless of lighting conditions [14,15,16]. The general suitability of these detectors has been tested in various studies and different setups so far. PID has already been used to measure the activity of free-ranging mouflons [17] or of piglets in open field experiments [18]. Additionally, data collection and analysis was conducted in stables for different types of animals, such as group-housed broiler chickens [19,20], laying hens [21], and particularly (weaner) pigs [3,14,15,16,22].

Behavioral data obtained in this way are of course rather rudimentary compared to those of the image-based innovations mentioned above. If, however, PID data can be interpreted sufficiently to make continuous, valid observations of animal behavior and deviations in animal welfare, the advantages of PIDs—low cost in terms of material and time due to easy handling, low process consumption, and good running performance [3,15]—could come into effect. Therefore, PIDs could be used as a simple but meaningful basic real time tool in the future for behavioral monitoring and assessment.

However, in the aforementioned PID studies, the activity data mostly referred to relatively large groups of animals or were undifferentiated for all animals in a compartment and less focused on smaller groups of pigs in a pen or in special (functional) areas within the pen. However, results of other studies that investigate specific behaviors indicate that behavioral observation can also provide important information in certain focus areas. For example, a change in the drinking behavior can indicate disease [23,24,25] or the adaptation to rising room temperatures [26] or increased activity on the enrichment material could indicate the outbreak of tail biting [27,28,29]. Thus, PIDs could possibly also be focused on specific areas of the pen to monitor behavioral changes. Furthermore, results of other studies already indicate how important and useful it is to relate measured barn climate parameters or indoor air quality such as airborne dust levels, heat, moisture, carbon dioxide or NH_3_ emissions to animal activity [16,21,30,31].

Therefore, this study focused on the following three aims:

- Further verification of PIDs for their suitability to record the activity of a group of fattening pigs within the pen in order to obtain information on behavioral changes during the day;
- Determining whether it is possible to obtain information on behavioral changes not only at group level in general but also in specific functional areas of the pen by focusing PIDs;
- Verification of the relationship between indoor environment parameters and the activity pattern of the focused animal group.

## 2. Materials and Methods

### 2.1. Animals and Housing

**Figure 1:**
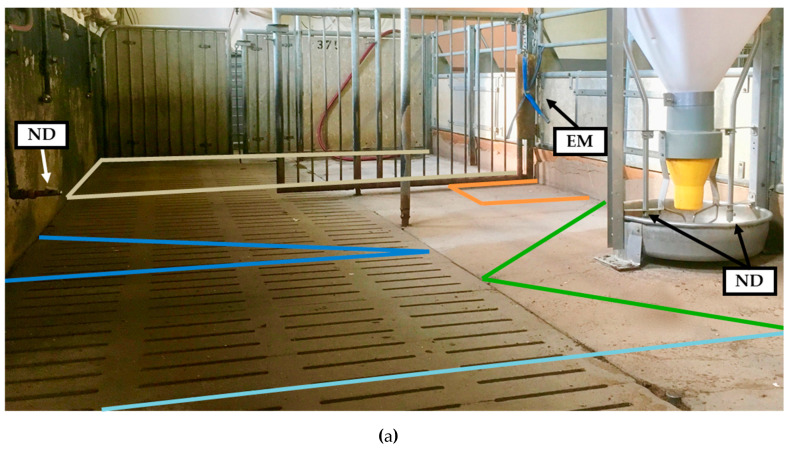

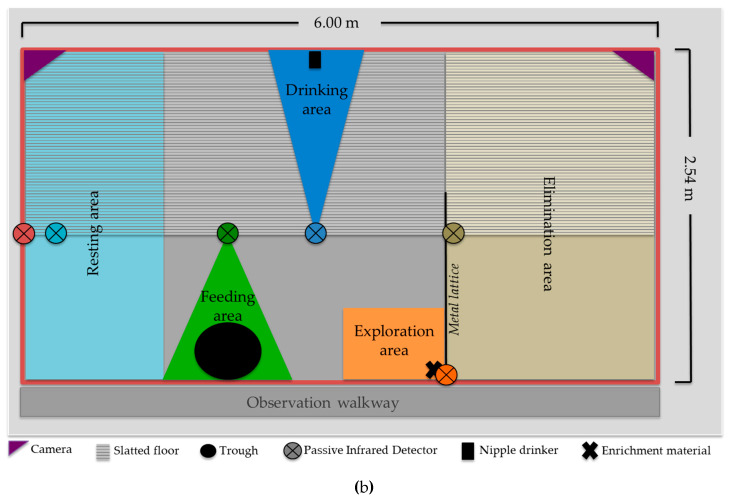
Image of the focus pen with indicated functional areas (**a**) for lying, drinking, eating, exploration and elimination, nipple drinker (ND) separate and included in the wet feeder and enrichment material (EM) as well as schematic floor plan (**b**) and positioning of the passive infrared detectors (PIDs) used for the corresponding functional areas as well as for measuring total activity of the animal group in the red framed area.

The trial was carried out on the educational and research center Frankenforst (Königswinter-Vinxel, Germany) of the Agricultural Faculty of the Rheinische Friedrich-Wilhelms-University Bonn. The experimental period covered one fattening cycle of 105 days and extended from March to July 2019 in a forced ventilated fattening stable. The measurements were focused on 11 weaned and docked pigs housed in a 6.00 × 2.54 m pen (see Figure 1) with partly slatted concrete floor and a metal lattice as the partition wall for additional structuring. The focus pen does not border on any side directly on another pen. It is located at the beginning of the compartment and on the other side, a delimited area of 2.00 m width, in which the data loggers were accommodated, separates it from the neighboring pen.

The pigs entered the pen with an average live weight of 23.0 kg and were slaughtered with an average carcass weight of 85.2 kg. Ten days before the end of fattening, one pig was excluded from the trial due to illness. The pigs were fed ad libitum on a wet feeder including two nipple drinkers (Figure 1a). In addition, water was supplied with a nipple drinker placed across the feeder. A cotton rope was offered as enrichment material and was exchanged once a week. In order to test the usefulness of PIR to assess behavioral interactions with a novel object in the form of an unknown enrichment material, at the end of the fattening on day 89, the rope was exchanged for an unknown object. This consisted of a combination of wood, metal chains, and plastic tubes.

### 2.2. Passive Infrared Detector and Functional Areas within the Pen

In total six PIDs were installed at a height of 1.36 to 1.76 m to measure the activity in five different functional areas plus the overall activity of the animal group itself. The observed focus areas were divided into their functions: the feeding area, the drinking area, the exploration area with enrichment material, the resting, and the elimination area (Figure 1). The PID (item no. 1362922, renkforce, Conrad Electronic SE, Hirschau, Germany) selected for this study provides an analogous signal. Due to modification of the detector signal, the activity is not displayed and stored in volts, but directly converted into a percentage. A voltage around 1 V corresponds to the resting state of the PIDs and an activity level of 0%, while a voltage of 10 V corresponds to an activity level of 100%. The basic voltage, which also differs between identically constructed sensors, was taken into account in order to compare the sensors independently. Beyond that, the PIDs have been equipped with a signal light for quick and easy testing of the functional readiness or to adjust the focus to specific areas. The measuring data were stored every minute by a data logger (ALMEMO^®^, Ahlborn Mess- und Regelungstechnik GmbH, Holzkirchen, Germany).

In accordance with the manufacturer’s specification, the PID has a detection angle of 360°. When mounted in a height of 2.50 m, it is able to detect movements in a distance up to 16.00 m. The range could be also affected by the mounting height, the temperature difference between the body and the background, the distance and size of the moving body, and by the intensity of movement.

**Figure 2:**
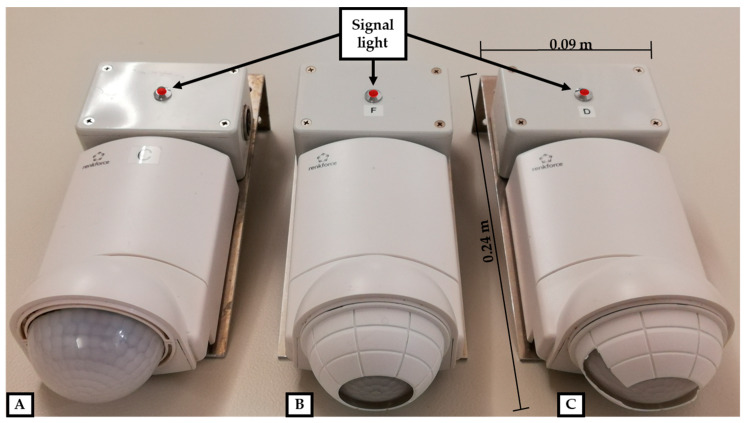
Passive infrared detectors with open (**A**), largely covered (**B**), and sector-wise covered (**C**) lens and signal light for functional testing.

In order to exclude certain areas from the detection or rather to focus the detector accurately, the lens can be partly covered as shown in Figure 2.

### 2.3. Visual Assessment as Reference Method

In order to evaluate activity data from the PID, the behavior of animals was recorded by video cameras for visual assessment. Two dome cameras were mounted on the compartment ceiling on both sides of the pen (see Figure 1b). Infrared radiators were installed underneath the cameras to be able to evaluate the behavior also at twilight or in darkness.

In contrast to the continuous recording of activity by the PID, the recording of behavior by cameras was done for certain focus days. To get a good overview of the entire fattening cycle, two control days were chosen at the beginning (fattening day 12 and 35), four in the middle (fattening day 40, 47, 61, and 68) and four days at the end (fattening day 75, 82, 89, and 96). The video material obtained was analyzed by assessing still frames in 10-min intervals over 24 h using a scan sampling method. Thus, 144 frames were analyzed per day. In order to be able to judge whether animals are moving, the still frames were also viewed one second before and one second after the frame. The behavior of the animals was generally divided into “inactive” or “active”. The inactive behavior was exclusively assigned to lying; active behavior, however, was further subdivided into standing, sitting, locomotion, drinking, eating, excretion, and exploration of enrichment material (Table 1). Drinking, eating, and exploration behavior was only classified as such if it was recorded in certain areas. For example, drinking was only classified as such at the separate nipple drinker and not at the ones in the trough. The level of activity was determined in percentage as the number of pigs per behavior in relation to the total number of animals. The overall activity arose from the sum of all single behavior patterns besides lying.

The visual assessment was performed by a single observer who was trained on the basis of the exact description of the defined behavioral subtypes (see Table 1) and video material independent of the experiment to avoid interpretation errors.

### 2.4. Climate Measurements

In addition to activity, important indoor environment parameters were recorded. To measure NH_3_ concentration in the stable air, one Polytron C300 (Dräger, Lübeck, Germany) equipped with an electrochemical gas sensor (DrägerSensor© NH_3_ AL; accuracy according to manufacturer: 1.5 ppm or ± 10% of the measured value) was installed in the middle of the pen at a height of 1.47 m. The data were stored every minute on a second data logger (ALMEMO^®^, Ahlborn Mess- und Regelungstechnik GmbH, Holzkirchen, Germany). Air temperature and relative humidity were measured with a Tinytag (Gemini Data Loggers Ltd., West Sussex, United Kingdom), which was also installed in the middle of the pen at a height of 1.86 m. The sensor stored the data in 10-min intervals.

In order to identify days with potential heat stress for fattening pigs, the Temperature Humidity Index (THI), or rather the THI_NOAA_ according to the definition of Vitt et al. [32] as a quantification parameter, was calculated with the subsequent equation:

(1) THI_NOAA_ = 0.81 T_DB_ + 46.3 + R / 100 (T_DB_ – 14.3)

where dry bulb temperature T_DB_ (= air temperature T), relative humidity R (%).

Following Vitt et al. [32] and St-Pierre et al. [33], a value of ≥72 was chosen as threshold with economic impact on fattening pigs.

### 2.5. Processing of Data and Statistical Analysis

Due to technical problems, no sensor data could be generated for fattening days 49 to 59. The available data were checked and corrected or invalidated when necessary due to signal interference of the detectors or other reasons. SPSS Statistics 25 (IBM, New York, NY, USA, 2017) was used to analyze the data. The distribution of data was tested with the Kolmogorov–Smirnov test. The quality of the linear relationship between the PID data and the visual assessment was verified using Spearman–Rho for not normally distributed data. The assessment of the strength of the effect—the effect size—of the determined correlations was carried out using the classification by Cohen [34]. The PID data were analyzed descriptively. For better representation and smoothing in graphics, the PID data recorded every minute were processed by calculating moving average values. In addition, the valid sensor data were averaged every 60 min and summarized for specific phases or time periods according to the respective question. The quality of the linear relationship between the corresponding variables of these “average days” thus obtained for each phase was verified using Spearman–Rho for not normally distributed data as well.

## 3. Results

### 3.1. Comparison between PID and Visual Assessment

The comparison of the visually recorded and calculated activity during fattening as well as the PID data recorded at the corresponding times are listed in Table 2.

For the measurement of activity by PID for the group activity, the activity at the trough, in the exploration area as well as in the resting area, strong correlations could be found compared to the visual assessment. In addition, the correlation between PID data for group activity and corresponding visual assessment seems to decrease over the course of fattening from r = 0.87 to 0.60. For the measurement of activity using PID at the nipple drinker and in the elimination area, often only significant correlations with low or medium effect strength could be detected.

**Figure 3:**
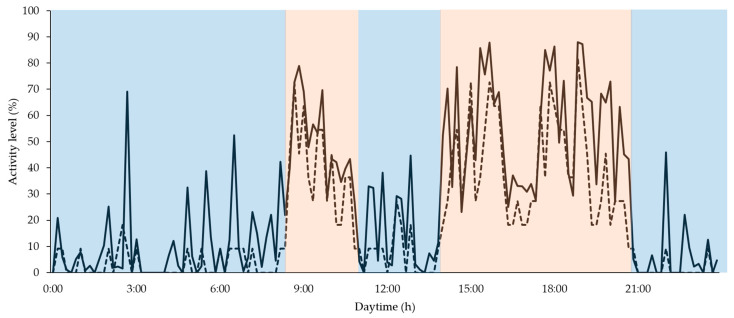
Group activity quantified on fattening day 47 using PID (continuous line) and visual assessment (dashed line) as well as exemplary division in calmer (blue background) and more active (orange background) phases.

Furthermore, the focus days were divided into calmer and more active phases depending on the measured average activity values of group PID and visual assessment (Figure 3).

In the statistical evaluation, no major difference in the strength of the effect for the correlation between PID and visual assessment could be found in dependence of the considered two phases, calm and more active, over all focus days (r = 0.54, *p* = 0.00, *n* = 848 and r = 0.56, *p* = 0.00, *n* = 592, respectively).

All focus days considered, the analysis shows that the PIDs generally overestimate activity (as derived from passive infrared activation), as compared to visual assessment of activity (as can be seen in Figure 3).

### 3.2. PID Combined with Behavioral Analysis

#### 3.2.1. Development of Group Activity over the Course of Fattening

**Figure 4:**
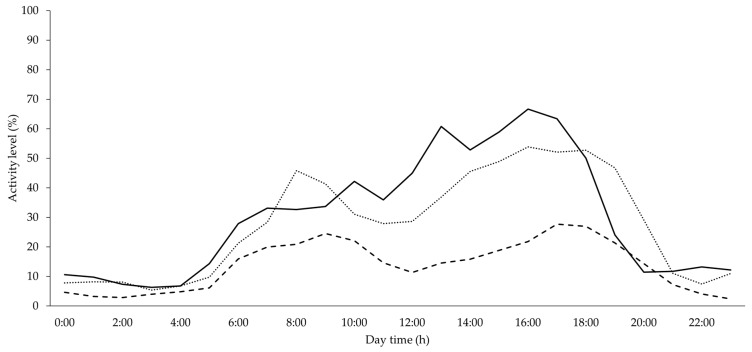
PID data on changes in group activity level dependent on daytime and fattening phase (continuous line = until fattening day 19, dotted line = fattening day 20–70 and dashed line = from fattening day 71).

Recording the animals’ activity over the entire fattening cycle provided the opportunity to illustrate the development of activity. As shown in Figure 4, the daily rhythm and the intensity of the animals’ activity change over the course of the fattening period.

In the case of the very young fattening pigs until fattening day 19, the overall activity level was high for the whole day with a number of peaks, the highest in the afternoon. Over time and starting approximately from the 20th fattening day, an activity profile evolves with one main peak in the morning and another, longer one in the afternoon, as well as resting periods at night and between daytime peaks. A biphasic course has adjusted. The type of activity or (defined) behavior shown by the animals does not differ between morning and afternoon peak. In the intermediate phase of the fattening period, the total activity level remained high. There was generally much less activity at night than during the day. During the course of fattening, the activity level decreased with increasing age and weight of animals. Toward the end of fattening, the biphasic pattern was yet visible, but the level of activity was noticeably reduced. Video analysis gives the impression that the younger fattening pigs generally move more, as well as faster and more erratically, although the data were not statistically analyzed to validate this observation. In addition, there was more free space available to them in relation to their body size.

#### 3.2.2. Use of Different Enrichment Material

**Figure 5:**
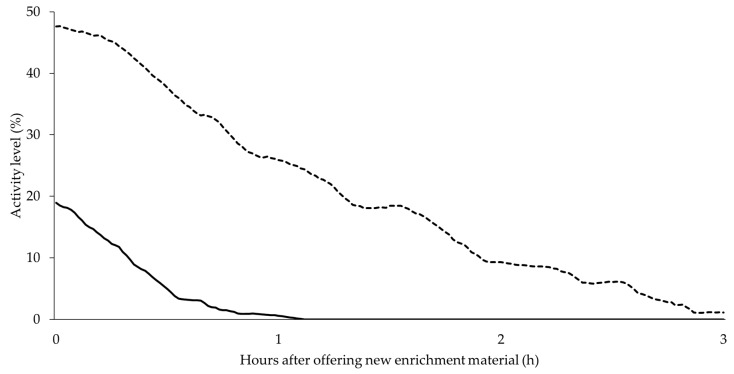
PID data on course of use and attractiveness of enrichment material after offering: well-known cotton rope (continuous line) versus unknown wooden toy (dashed line) at the end of the fattening.

Using PID in the exploration area, the utilization and attractiveness of enrichment material could be recorded and assessed. In general, activity in the exploration area decreased significantly over the course of fattening (r = −0.79, *p* = 0.00, *n* = 93). Toward the end of the fattening period, if the pigs were offered a fresh cotton rope, interest increased at first in contrast to the older one but decreased relatively quickly after offering as shown in Figure 5. However, the same figure illustrates that the interest in a new and unknown object is higher from the beginning and lasts longer.

**Figure 6:**
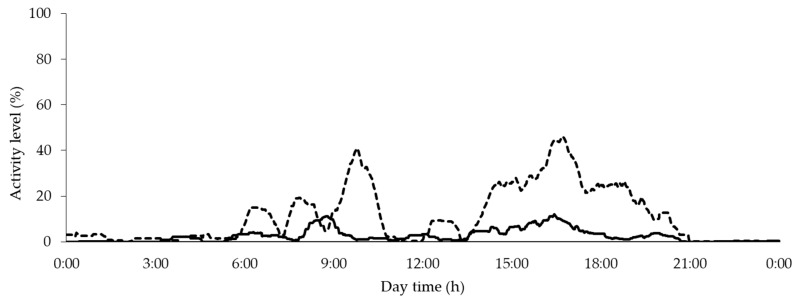
General course of exploration measured by exploratory PID during the day with two activity peaks as well as comparison between well-known cotton rope (continuous line) versus unknown wooden toy (dashed line) with one-week interim.

Comparing the activity course of two whole days with different enrichment materials, the result was as follows: although the animals generally become more inactive with increasing age and weight, the supply of an unknown wooden toy at the end of the fattening period can considerably increase use (Figure 6).

The agreement of the exploratory PID data with visual assessment showed that the measured activity was indeed employment behavior. The significant correlations for the day with the cotton rope and the wooden toy was r = 0.57 (*p* = 0.00, *n* = 144) and r = 0.88 (*p* = 0.00, *n* = 144), respectively. The activity level measured by the detector for the wooden toy was on average about 14% higher than for the cotton rope.

As well as in the assessment of group activity, the use of enrichment material had its peaks in the morning and afternoon, while the activity in the afternoon was more pronounced.

### 3.3. PID Combined with Climate Analysis

#### 3.3.1. Indoor Climate

**Figure 7:**
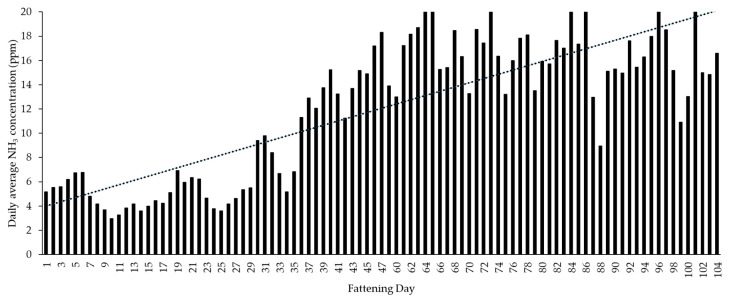
Statistical relationship between fattening days and daily average NH_3_ concentration (*n* = 93) measured above the focus pen. Significant increase (r = 0.77; trend line see dotted line) in NH_3_ concentration over the whole fattening period.

Over the whole test period, the average temperature and relative humidity measured above the focus pen was 21.47 °C ± 3.29 and 50.99% ± 8.24. NH_3_ concentration was on average 12.05 ppm ± 6.94. As shown in Figure 7, NH_3_ concentration increased significantly throughout the fattening period (r = 0.77, *p* = 0.00, *n* = 93).

#### 3.3.2. Activity as a Function of THI

**Figure 8:**
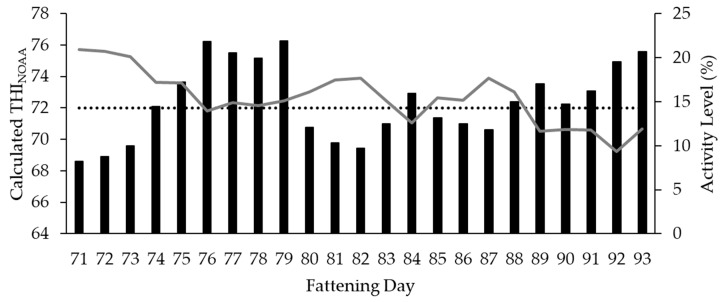
Comparison with reference to daily average values of the group activity measured by PID (continuous line) with the calculated THI_NOAA_ (bar chart) in consideration of a threshold ≥72 (dotted line). Only an extract of the fattening period with corresponding thresholds is presented.

The results indicate that when pigs suffered from heat stress, they changed their behavioral pattern toward a reduction of activity (Figure 8).

For the total of 82 fattening days, during which all relevant data were available, a significant correlation between group PID as well as trough PID and THI_NOAA_ could be determined (r = −0.80 (*p* = 0.00, *n* = 82) and r = −0.83 (*p* = 0.00, *n* = 82), respectively). The available results indicate that the animals reduced not only their activity per se, but also the activity at the trough and thus the feed intake with increasing THI.

#### 3.3.3. NH_3_ Concentration Profile as a Function of Activity

**Figure 9:**
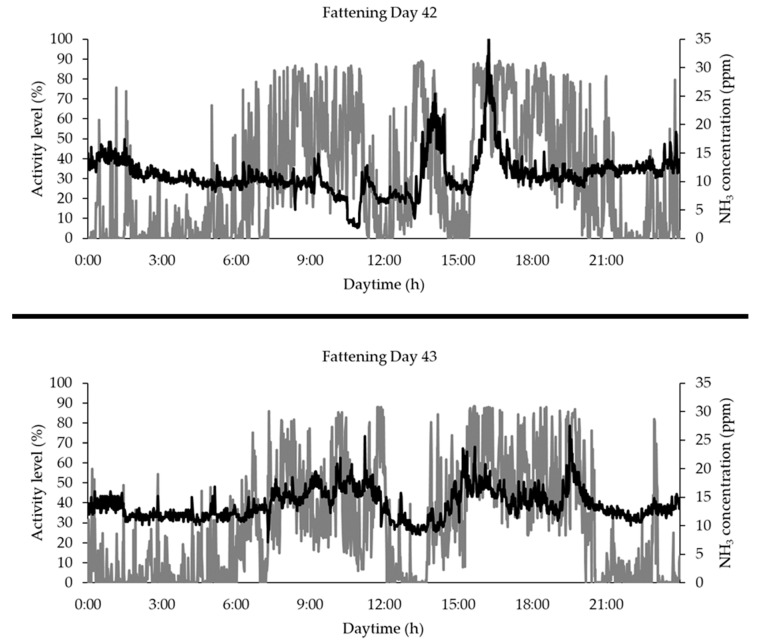
Graphical representation of the different relation between group activity measured by PID (grey line) and NH_3_ concentration (black line) on fattening day 42 (r = −0.01, *p* = 0.71, *n* = 1440) and 43 (r = 0.58, *p* = 0.00, *n* = 1440).

For the sum of the evaluable data (*n* = 128,561), no relevant correlation between group activity and NH_3_ concentration could be determined. If the available data are summarized in hourly averages and the entire measurement period is divided into two phases as they result from Figure 7—phase with low NH_3_ concentrations (FD < 35) and phase with higher NH_3_ concentrations (FD ≥ 35)—no consistent correlation between group activity and NH_3_ concentration can be found here either. However, there are considerable differences between the individual fattening days with regard to the context under examination. For example, there is no relevant correlation between the two parameters on fattening day 42 (r = −0.01, *p* = 0.71, *n* = 1440). For the following fattening day 43, however, a significant correlation of r = 0.58 (*p* = 0.00, *n* = 1440) can be demonstrated (see also Figure 9).

## 4. Discussion

### 4.1. Comparison between PID and Visual Assessment

The correlation between PID data and visual assessment with regard to the measurement of group activity was very strong with correlations from up to 0.87 (Table 2) and comparable to that of other studies despite differences in definition and assessment/scoring of behavior and activity [14,17,18]. For comparison, in Besteiro et al. [14], the correlation determined for the dataset was 0.86. This is comparable to the correlation of 0.92 determined by Ott et al. [5], who compared automated video analysis of pixel changes with human observation of activity in pigs. Thus, the estimation of the group activity level is possible with both PID and automated video analysis [14]. Compared to the activity measurement by automated video analysis, however, the data evaluation and processing has so far been considerably simpler, and the activity measurement of PIDs is independent of the time of day or the available light, which can sometimes restrict video-based behavior evaluation.

Furthermore, in Puppe et al. [18], significant strong correlations between behavioral observation by an observer and the PID technique were found. In addition, it was shown that PID measures data more reliably in terms of objectivity, especially in situations where the observer’s assessment is influenced more by expectations of certain events than by the actual events or behavior of the animals themselves [18]. In addition to the objectivity of PID, this technique also allows a largely undisturbed recording of behavior compared to direct observation, the costs of which are also higher for manpower and technical investment [17].

Comparing the correlations of the present study between group PID data and visual assessment over the 10 focus days, a decreasing comparability with proceeding fattening could be determined, as was also found by Besteiro et al. [14]. They justified this correlation with higher and more homogeneous temperatures with increasing live weight of the animal, which presumably influences PID measurement based on temperature differences [14]. However, in comparison to the results of Besteiro et al. [14], no significant differences in the strength of the correlation between calm and more active phases could be found in the present study. The differences may be due to the fact that the day itself was not strictly divided into night and daytime in the present study, but the phases were divided into rest and activity phases depending on the measured activity. Thus, for example, resting phases during day and night were combined for the correlation analysis; on the other hand, (total) activity was not classified exclusively via feeding and playing behavior as in Besteiro et al. [14]. Other behaviors, such as undirected sitting or standing, which also influence the PID signal strength, may have been less frequent at night than during the day and thus led to stronger correlations between visual assessment and PID in Besteiro et al. [14].

Comparable to the results of Besteiro et al. [14], the present experiment also showed that PIDs generally overestimate activity (as derived from passive infrared activation), as compared to the visual assessment of activity. A possible reason might be that the detector measured the intensity of activity, while the human observer determined activity by the number of animals that pursued a behavior except lying. Because the single behaviors of an animal did not always trigger the same level of activity, the detector received impulses of varying intensity. Therefore, the PID method was more sensitive than human observation and visualized the actual level of activity more precisely.

### 4.2. PID Combined with Behavioral Analysis

In general, activity data of the group PID show a circadian rhythm, which is considered as typical for pigs and which has already been described in other studies, partly also by PIDs [3,14,15,35,36,37]. In the course of fattening, an activity profile emerged with two activity peaks, one in the morning and a more pronounced one in the afternoon. Between these two activity peaks during the day and in the evening and night hours, resting phases can be observed, which are only partially interrupted by individual animals rising to drink or to change their position. Other studies have also described that a certain circadian rhythm is formed or consolidated over time, which is then maintained until the end of the observation period [3,15,35]. As in other studies, at the end of the experimental phase, a biphasic rhythm with two peaks per day was observed [3,15,38,39], which in Hillmann et al. [35] was also found in the context of salivary cortisol measurement—a parameter for stress and non-stress-related physiological action with effects on animal welfare [40]. As with Saha et al. [38] or Ni et al. [15], in the present experiment, the second daily peak in the afternoon proved to be more intensive and/or longer-lasting than the morning peak.

Based on the knowledge about the activity profile typical for pigs in general and about the individual profile of the pen or the compartment depending on feeding management or similar, in future, the PID data could be used as a basis for early warning systems in case of deviations. Changes in resting and activity phases—be they related to course, frequency or intensity—could provide important information on animal welfare. For example, changes in the typical daily rhythm and a reduction in activity in general could appear in the case of disease [41] as well as triggered by heat stress [42]. The latter is also indicated by our own results of reduced activity in general and at the trough with increasing THI values (see Figure 8). In addition, changes in the typical daily rhythm in the sense of an increase in activity could provide early warning of the outbreak of tail biting [28,29,43,44]. Furthermore, the activity data of a group of animals could be used for control purposes in order to observe the development of fights for establishing a social hierarchy [45] at the beginning of housing in or moving pigs. This could be used to monitor whether the frequency of fights decreases after usually a few days [46] and, when intensive phases of fighting occur during the day, which could be mitigated by the administration of additional enrichment material to reduce possible injuries to the animals. However, not only group-based activity data but also data related to specific focus areas such as trough or drinker can provide important information for the assessment of animal welfare. Therefore, the suitability of the PIDs for activity detection in certain functional areas was also investigated in the present experiment.

Due to strong correlations, the data of the PIDs in the resting, feeding, and exploration area of the pen in focus proved to be meaningful for the expected behavior corresponding to the specific functional area. The partly very weak correlations for the PIDs in the drinking and elimination area are probably mainly due to deficiencies in the position of the functional areas themselves and the dual use. Although an increased amount of space per animal compared to practice has already been implemented in order to allow the animals an improved division of the pen into separate functional areas, not all circumstances could be optimized for later evaluation. For example, the nipple drinker PID did not primarily record drinking animals or animals at the drinker, but frequently animals on their way from the elimination area to another functional area such as the trough area. Furthermore, the focused drinker was not the only source of water for the animals in this pen. As fattening progressed, an increase in the use of the drinker at the wet feeder was observed. Presumably, the animals use the possibility of increased water intake per unit of time at the feeder compared to the focused nipple drinker [25] in order to meet their increasing total water consumption rate during fattening [24]. This is because the nipple drinkers included in the wet feeder not only allow the animals to take in water directly, but also to fill the trough with it and thus take in water from an open surface, regardless of the current water flow rate.

Due to the good correlations between PID and visual assessment compared to drinking and elimination area, the activity data for the trough, exploration, and resting area in the focus pen could in future be directly included in the assessment of animal welfare.

The assessment of activity at the trough requires the following knowledge: Pigs are synchronous eaters [47], who also show a biphasic feed intake rhythm with two peaks when fed ad libitum [48]. Similar to the results of de Haer and Merks [48], the wet feeder PID data in the focus pen show a peak in the morning and a higher or rather longer-lasting peak in the afternoon. A changed feed intake behavior can have different influencing factors. On the one hand, temperature not only has an influence on water intake [26] but can also lead to a reduction in feed intake [42]. In the present experiment, a reduced activity at the trough was observed with increasing THI. Collin et al. [26] and Quiniou et al. [49] also describe a decrease in feed intake associated with a shorter daily consumption and ingestion time. A general shift in feed intake times during the course of the day [26] or a change in the daily number of meals [26,49] due to high ambient temperatures could not be observed. Another factor may be diseases that adversely influence activity at the trough and the appetite of animals [41]. As a third factor, the animal/feeding place ratio and therefore the competition for feed can also influence activity and the circadian pattern and thus indicate too much stress at feeding times. Thus, Nielsen et al. [50] showed differences in feeder occupation during the day depending on group size. As in the experiment, the feed intake was distributed over two peak periods, one in the morning and a second in the evening [50]. In contrast to the unchanged morning peak, in the afternoon, the feed intake was distributed over a longer period of time and partly extended into the evening hours when group sizes were increased [50]. Hyun and Ellis [51] also showed an increase in the proportion of time the feeder was occupied with increasing group size.

A decrease in activity on the enrichment material can also indicate deficiencies in husbandry or rather management. Play behavior in general is considered to be an important indicator of positive emotions and indicates welfare [1,52,53], as it is only expressed when all other needs are met and environmental conditions are ideal for the animal [1,54]. In the experiment itself, however, only the play with enrichment material was recorded and evaluated, not play behavior between conspecifics or other furnishings. A decreased activity in the exploration area was observed during the course of fattening. In the experiment, the activity could be increased for a short time when changing the cotton rope as a generally already known material. However, if the pigs were offered a completely unknown item at the end of the fattening period, an increased duration of activity shortly after the addition and an increased activity during the course of the day in general was observed. The added value of the information the PID could provide by activity measurement in the exploration area is that the farmer is able to change the current material as soon as it loses its long-term attractiveness and it therefore no longer fully serves its purpose. Such a habituation to the offered material and thus a loss of attractiveness can occur very quickly within a few days [55].

Moreover, increase in activity on the enrichment material without having previously exchanged it could also indicate the outbreak of tail biting [27,28,29].

Overall, the results of the PIDs focused on specific areas confirm the statement of Besteiro et al. [14] that affixing and focusing of the PIDs is decisive for meaningful results [14]. Weak correlations for certain functional areas and corresponding behavior have already been determined by optimized pen design or space available per animal. Thus, testing the PIDs in functional areas under practical conditions should be the next important step to further investigations on the suitability of PIDs.

### 4.3. PID Combined with Climate Analysis

With regard to climate parameters, the focus of this study was primarily to investigate the relationship between NH_3_ concentration and overall activity of the animal group. Taking into account the entire fattening period, the results show a clear correlation between NH_3_ and the fattening day (Figure 7), as already shown by Aarnink et al. [30] or Jeppsson [56]. As the fattening progresses, the measured NH_3_ concentration increases. The fluctuations or partly abrupt reductions as seen in Figure 7, for example, on fattening days 42, 88 or 99, are probably attributable to the ventilation or cleaning management, which was not recorded in this experiment. However, it turned out that the functional areas were used differently by the pigs depending on the air temperature inside the barn and the growing size of the pigs. With rising inside air temperatures, the animals increasingly lay down on the slatted floor. In addition, the elimination area was extended to the concrete floor area of the pen and thus to other functional areas (exploration and feeding area). The extension of the elimination area resulted in the animals also depositing feces and urine in the floor area below the NH_3_ sensor. To avoid heat stress and further pollution of pen and pigs by wallowing, in addition to increasing the ventilation rate, the solid areas were regularly cleaned with water. Especially the cleaning of the pen and thus the removal of feces probably led to this partly noticeable reduction of NH_3_ concentration, which only lasted for a short time. A similar cause–effect relationship between cleaning and the reduction of NH_3_ emissions was shown by Aarnink et al. [30].

A consistent correlation between NH_3_ concentration during the course of a day and the animal activity on the basis of each individual fattening day of the fattening period [30,56] could not be proven in this study. Figure 9 shows how strongly the correlation between NH_3_ and activity differs between two individual fattening days. While on fattening day 43, for example, the diurnal character of NH_3_ concentration is clearly recognizable depending on the animal activity, a comparable correlation is not seen the day before. On the one hand, the proven higher correlation found by Jeppsson [56] could be attributable to the investigated deep litter system. On the other hand, NH_3_ concentrations in the barn air are significantly influenced by a number of other factors (e.g., ventilation rate, outside air temperature, liquid manure management, cleaning management, and barn hygiene) [57,58,59,60], which unfortunately could not be measured and evaluated in this study.

The effects of increasing THI levels and thus of temperature and relative humidity on activity have already been described in the context of group and trough activity measurements. Nevertheless, the following should be pointed out in connection with the temperature: Air temperature above 28 °C should be avoided because it leads to heat stress for the animals [32]. Heat stress degrades animal welfare as pigs are not able to sweat to the appropriate extent and suffer from high temperatures [42]. To prevent these negative effects of temperature, the use of PIDs could help to recognize behavioral changes early and take countermeasures such as evaporative cooling with cooling pads [32] to improve animal welfare.

### 4.4. Outlook for Future Application Possibilities in Practice

Overall, it can be summarized that PIDs and the activity data they generate in combination with other information would meet the demand not only to determine the current welfare status as a monitoring system, but also to be able to indicate potential risks at an early stage [2]. PIDs can be used to monitor general behavioral changes that may be related to factors or circumstances such as heat stress, disease or tail biting that may have a negative impact on animal welfare. However, further development of the output and analysis of PID data is needed to improve the usability and gain of information for the farmer. A meaningful processing and presentation with the help of, for example, algorithmic processing or supported by artificial intelligence is to be aimed at for practical use as well as the increased fusion of data from various sensors [61].

In future, low-cost and easy to handle PIDs could be used in two very different ways to complement and improve the assessment of animal welfare: for self-monitoring and as an on-farm consulting tool.

On the one hand, PIDs could be used for internal monitoring of livestock (even with large numbers of animals) to supplement continuous assessment with regard to animal-welfare-relevant indicators and precise documentation of so far mainly resource- and management-based indicators such as temperature, relative humidity or NH_3_ concentration. In this way, appropriate animal welfare on farm can be more comprehensively evaluated and monitored by the animal owner themselves. As a decision support system, it can thus indicate changes in animal behavior at an early stage, to which the farmer can then react according to the situation, even before there is any actual impairment of animal welfare, e.g., through the outbreak of tail biting or heat stress. However, PIDs cannot and should not replace the daily visual inspection of animals by the farmer.

On the other hand, a profile of the group activity or the activity in certain focus areas obtained by the PIDs can also be used as a supplement to (already existing) animal welfare assessment protocols for evaluation or consultation in the context of a Weak Point Analysis. In addition to the usual resource- and management-based indicators, more attention is already being given to assessment using animal-based indicators. In addition to the assessment of health (using slaughter data) and the status quo assessment of phenotype (via BCS, manure on the body or the like) or behavior by novel object test, assessment of exploration behavior, etc., the behavior of animals could thus be incorporated even more meaningfully and above all much more objectively into the assessment of animal welfare by means of PIDs as a basic real time tool.

## 5. Conclusions

The experiment showed that PIDs are suitable for recording the activity of a group of fattening pigs in a pen. The activity profile obtained over the day, over a specific phase or over the whole fattening period allows initial conclusions to be drawn about resting, stress, and activity phases, which can be used combined with other factors to assess animal welfare. In addition, it was shown that activity profiles could be obtained in certain clearly definable areas of the pen such as the trough, the resting or the exploration area with the help of PIDs. The results of the present investigation based just on one pen indicate the following: These profiles in combination with each other and with further information on group activity as well as data on, for example, the temperature in the compartment can improve the assessment of the situation in the pen or barn and enable the farmer to react to behavioral changes at an early stage.

In future, low-cost and easily manageable PIDs should not only be used in the context of scientific questions but should also be used for operational self-monitoring of animal welfare or as part of an on-farm consulting tool. They can be used for an entire compartment, a pen or for specific, definable functional areas within the pen. Since in the present study, only one pen serves as the basis for the results of focusing PIDs, further investigations must show the extent to which stables or compartments and corresponding focus areas must be equipped with PIDs in order to be useful for the purpose of obtaining information and to be profitable at the same time.

## Figures and Tables

**Table 1:** Allocation and definition of the observed behavior.

General Division In	Subtype	Description	Place-Bound Behavior Assessment?
Inactive	Lying	Pig lying in prone or lateral position without performing any activity	No
Active	Standing	Pig stays longer than 1 sec on the spot, the floor is only touched with the feet and possibly the snout	No
	Sitting	Forefeet and hindquarters of the pig have ground contact at the same time	No
	Locomotion	Movement at any speed and in any direction	No
	Excretion	Pig urinates and/or defecates	No
	Drinking	Pig is in a standing or sitting position and has the nipple drinker in its mouth	Yes, nipple drinker/drinking area
	Eating	Pig is in standing or sitting position at the feeder and keeps its muzzle in the trough	Yes, wet feeder/feeding area
	Exploration of enrichment material	Pig chews, bites, licks, drags or works with its snout on the enrichment material (regardless of body position)	Yes, enrichment material/exploration area

**Table 2:** Statistical analysis for group activity as well as activity in the focus areas calculated by visual assessment and measured by passive infrared detectors (PIDs). One-hundred and forty-four data pairs were available for evaluation on each of the ten focus days.

(Focus) Areas and for this Area Expected or Rather Defined Behavior Measured with Corresponding PID vs. Visual Assessment	Fattening Day (with Visual Assessment)									
	12	35	40	47	61	68	75	82	89	96
Group PID + active behavior	0.87 **	0.83 **	0.85 **	0.86 **	0.84 **	0.79 **	0.78 **	0.71 **	0.80 **	0.60 **
Wet feeder PID + Eating	0.73 **	0.70 **	0.80 **	0.80 **	0.69 **	0.71 **	0.61 **	0.76 **	0.74 **	0.62 **
Nipple drinker PID + Drinking	0.30 **	0.35 **	0.17 *	0.20 *	0.26 **	0.16	0.21 *	0.15	0.24 **	0.24 **
Exploratory PID + Exploration	0.43 **	0.59 **	0.54 **	0.24 **	0.75 **	0.36 **	0.60 **	0.57 **	0.88 **	0.47 **
Resting PID + Lying	−0.80 **	−0.79 **	−0.83 **	−0.79 **	−0.76 **	−0.70 **	−0.73 **	−0.65 **	−0.70 **	−0.47 **
Elimination PID + Excretion	0.28 **	-	0.19 *	0.24 **	0.24 **	0.08	0.31 **	0.14	0.12	0.12

* *p* < 0.05 and ** *p* < 0.01.
